# Human cytomegalovirus major immediate early transcripts arise predominantly from the canonical major immediate early promoter in reactivating progenitor-derived dendritic cells

**DOI:** 10.1099/jgv.0.001419

**Published:** 2020-05-04

**Authors:** Rebecca Mason, Ian J. Groves, Mark R. Wills, John H. Sinclair, Matthew B. Reeves

**Affiliations:** ^1^​ Institute of Immunity & Transplantation, University College London, Royal Free Campus, London NW3 2PF, UK; ^2^​ Department of Medicine, University of Cambridge, Addenbrooke’s Hospital, Cambridge, CB2 0QQ, UK

**Keywords:** cytomegalovirus, chromatin, gene expression, major immediate early promoter, reactivation

## Abstract

Human cytomegalovirus latency and reactivation is a major source of morbidity in immune-suppressed patient populations. Lifelong latent infections are established in CD34+progenitor cells in the bone marrow, which are hallmarked by a lack of major lytic gene expression, genome replication and virus production. A number of studies have shown that inhibition of the major immediate early promoter (MIEP) – the promoter that regulates immediate early (IE) gene expression – is important for the establishment of latency and that, by extension, reactivation requires reversal of this repression of the MIEP. The identification of novel promoters (termed ip1 and ip2) downstream of the MIEP that can drive IE gene expression has led to speculation over the precise role of the MIEP in reactivation. In this study we show that IE transcripts arise from both the MIEP and ip2 promoter in the THP1 cell macrophage cell line and also CD14+monocytes stimulated with phorbol ester. In contrast, we show that in *in vitro* generated dendritic cells or macrophages that support HCMV reactivation IE transcripts arise predominantly from the MIEP and not the intronic promoters. Furthermore, inhibition of histone modifying enzyme activity confirms the view that the MIEP is predominantly regulated by the activity of cellular chromatin. Finally, we observe that ip2-derived IE transcription is cycloheximide-sensitive in reactivating DCs, behaviour consistent with an early gene designation. Taken together, these data argue that MIEP activity is still important for HCMV reactivation but ip2 activity could play cell-type-specific roles in reactivation.

## Introduction

A hallmark of human cytomegalovirus infection is the establishment of a lifelong latent infection of the host [1]. In healthy individuals these infections are asymptomatic but in immune-suppressed populations primary infection, re-infection, or reactivation from latency can be a major source of morbidity [2]. As such, understanding the molecular mechanisms that underpin HCMV reactivation has long been considered to have important applications for the development of novel anti-viral therapies [3].

The establishment of HCMV latency is dependent on the eventual silencing of immediate early (IE) gene expression from the major IE promoter (MIEP). Given the central role IE gene expression plays in productive infection it has long been considered that an induction of previously silenced IE gene expression represents the first stage in the reactivation from latency. Consequently, the differential regulation of the MIEP is likely to be a key molecular mechanism governing this switch. Consistent with this, the MIEP is responsive to myeloid differentiation signals, *per se*, as well as a number of inflammatory stimuli, which are known to promote virus reactivation [4–10]. Furthermore, the transfected MIEP displays increased activity in cells that are permissive for HCMV lytic infection but reduced activity in cells which support latency [11]. Studies of natural latency have shown that the MIEP is associated with host chromatin and, importantly, the post-translational repressive or activatory modifications associated with the histones bound to the MIEP correlate with the expression of IE genes during latency and reactivation, respectively [12]. Finally, mutation of transcription factor binding sites within the MIEP have been demonstrated to impact on HCMV reactivation in a cell type and model-dependent manner [13–16]. Taken together, these data point towards the MIEP being important during all phases of HCMV infection.

A recent report using an experimental model of HCMV latency in CD34+cells and the CD14+monocytic THP1 cell line has challenged this view and suggested that induction of IE gene expression during reactivation is triggered by alternative promoters to the canonical MIEP that are encoded within an intron of the MIE reading frame (designated ip1 and ip2) [17]. The activity of these promoters was reported to be non-essential during lytic infection of fibroblasts *in vitro* and thus it was suggested they could have reactivation-specific function during reactivation [17, 18]. Indeed, similar hypotheses have been suggested for the NF-kB, CREB and AP-1 transcription factor binding sites in the canonical MIEP whereby they exert the greatest impact on efficient viral reactivation [13–16, 19, 20].

In our ongoing studies of HCMV reactivation, which focus on the biology of dendritic cells (DCs) in this process, it became evident that THP1-derived macrophages did not respond in an equivalent manner to DCs when challenged with inhibitors of HCMV IE gene expression [21]. Thus, in light of the recent report of an alternative promoter driving major IE expression in certain myeloid cell types [17], we investigated whether these differences could be explained by model-specific usage of different viral promoters. Here we show that, in agreement with a previous report [17], the induction of IE gene expression from an alternative major IE promoter was detectable in latently infected THP1 cells stimulated with phorbol ester although there was also clear evidence of concomitant transcription from the canonical MIEP. Furthermore, in contrast to observations in THP1 cells, we also observed that IE transcription in reactivating DCs was predominantly MIEP derived, suggesting a cell-type-specific role for the different canonical (MIEP) and non-canonical (ip1 and ip2) major IE promoters. Intriguingly, an inhibitor of histone acetyltransferase (HAT) activity that selectively inhibited IE gene expression in reactivating DCs versus THP1 cells [21] was also demonstrated to inhibit the canonical MIEP, but not the alternative IE promoter, in the reactivating DCs. Finally, the IE transcription originating from the alternative promoter in DCs (but not the MIEP-derived IE transcripts) was abolished in the presence of cycloheximide. Thus, at least in DCs, ip2-derived transcription is cycloheximide-sensitive, behaviour consistent with an early gene. Taken together, these data argue that IE transcription in macrophages and DCs generated *in vitro* from primary myeloid progenitors using well-established protocols is MIEP-derived. Importantly, both macrophages and DCs have been demonstrated as important sites of HCMV reactivation *in vivo* [22, 23] – cell types on which the models of reactivation used here are based – arguing that MIEP activity is important IE gene expression in established sites of HCMV reactivation.

## Methods

### Ethics statement

The collection of venous blood samples from anonymous donors was approved and performed in accordance with established guidelines for the handling and processing of said tissue by the UCL and Cambridge Local Research Ethics committees. All studies with human material abide by Declaration of Helsinki principles.

### Viruses and inhibitors

The HCMV isolates Merlin and TB40/e were purified from infected human retinal pigment epithelial cells using sorbitol gradients as previously described [22]. Viruses for these studies were characterized by their ability to infect primary dendritic cells to assay myelo-tropism – routinely, virus preparations infected 10–20 % DCs when used at an m.o.i. of 5 calculated on fibroblasts.

To test for inhibition of HCMV reactivation, histone acetyltransferase inhibitors (HATi) C646 and MG149 were added 1 h prior to IL-6 stimulation. p300: C646 (SIGMA; 25 uM), MOZ: MG149 (Axon Medchem; 25 uM) or cells were treated with DMSO solvent control.

Inhibition of histone deacetylase activity was achieved using histone deacetylase inhibitor (HDACi) Romidepsin (3 nM), which was dissolved in DMSO. Inhibition of protein translation was achieved using cycloheximide (50 ug ml^−1^; SIGMA), which was added 6 h prior to stimulation of reactivation.

### Latency and reactivation experiments

CD14+monocytes were isolated from apheresis cones (NHSBT, Cambridge or NHSBT, Colindale, London) by MACS CD14+positive cell separation (Miltenyi Biotec) before seeding on plastic and subsequent feeding with X-VIVO-15 supplemented with 2 mM l-glutamine. After 24 h, cells were infected with HCMV Merlin or TB40/e at an m.o.i. equivalent to 5 on human foreskin fibroblasts. After a further 5 days, cells were then treated as follows for different experiments: 24 h with HDACi (Romidepsin, 3 nM), an equivalent dilution of DMSO, or PMA (20 nM); 4d with M-CSF/IL-1β (20 and 10 ng ml^−1^, respectively); or 6d with GM-CSF/IL-4 (both 1000 U ml^−1^) and a further 6–24 h with LPS (500 ng ml^−1^) or IL-6 (500 ng ml^−1^) to promote reactivation.

THP1 cells were infected at an m.o.i.=5 and cultured in 2 % RPMI-10 for 5 days incubated with PMA (20 nM) for 48 h to promote differentiation to a macrophage-like phenotype.

### Nucleic acid isolation and analysis

To isolate RNA, cells were washed once with PBS before direct harvest of samples with RLT buffer (Qiagen). RNA extraction was then performed as per the manufacturer’s instructions using the RNeasy Mini Kit (Qiagen). RNA (or RNA from historic studies) was converted to cDNA, then amplified by Sybr green quantitative real time-PCR using previously published gene-specific primers [5, 17]: UL123 5′- GCG CCA GTG AAT TTC TCT TC and 5′- ACG AGA ACC CCG AGA AAG ATG 3′; MIEP-derived IE 5′-TTG ACC TCC ATA GAA GAC AC 3′ and 5′-AGG ACT CCA TCG TGT CAA GG 3′; ip2-derived (UTR70) 5′-TAG CTG ACA GAC TAA CAG AC 3′ and 5′- AGG ACT CCA TCG TGT CAA GG −3′; 18S 5′- GTA ACC CGT TGA ACC CCA 3′ and 5′- CCA TCC AAT CGG TAG CG – 3′. Relative expression was analysed using the 2delta delta Ct method comparing control with test sample. To express absolute values in the qPCR analyses 2delta Ct was used to represent signal.

### Statistical analyses

The Mann–Whitney U test was applied to test for significance between the means as a non-parametric distribution was assumed. Statistical analyses were only applied if *n*>2. All scatter plots and bar charts depict the mean and one standard deviation from the mean. Significance was assumed if *P*<0.05.

## Results

### MIEP-derived transcripts predominate in reactivating dendritic cells but not THP1 cells

**Fig. 1.**
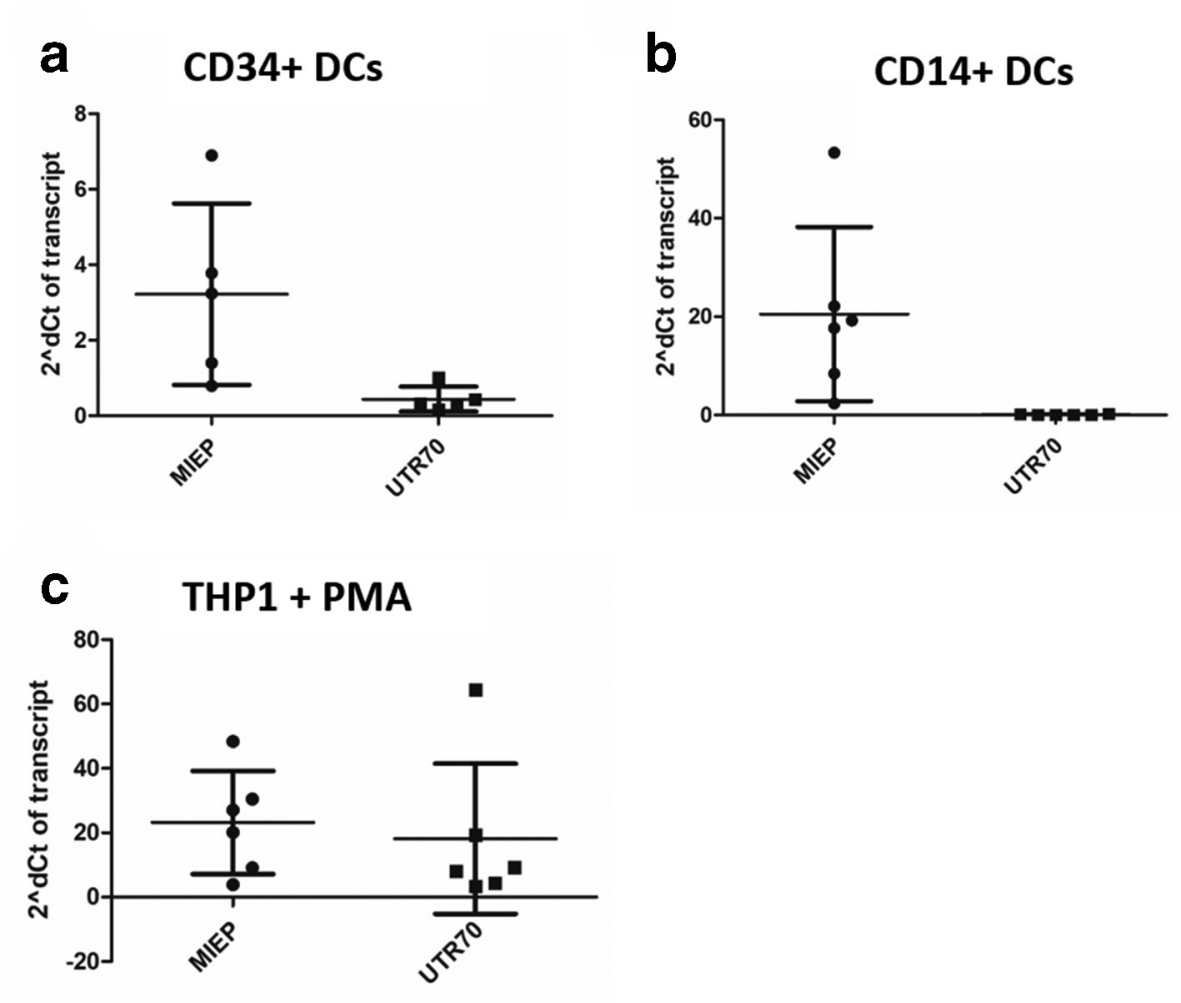
MIEP-derived transcripts predominate in DCs but not THP1-derived macrophages (a–c). Experimentally latent CD34+cells (a), CD14+monocytes (b) or THP1 cells (c) were differentiated to DCs (a and b) or macrophages (c) and RNA was analysed by qRT-PCR with primers that amplified, MIEP-derived IE RNA (MIEP), ip2-derived UTR70 RNA (UTR70) and cellular 18S gene expression 16 h post stimulation with IL-6. Gene expression is reported using the 2deltaCT method from five independent experiments.

To investigate the origin of IE gene expression in reactivating cells we analysed historic samples [16, 24] available from previous studies of experimental HCMV latency (Fig. 1). To do this, we took advantage of previously published primers used to investigate the origin of HCMV IE transcripts during lytic infection [18]. Primers that detect MIEP derived and ip2-derived (UTR70) transcripts were used to amplify cDNA by qPCR (Fig. 1a–c).

Interestingly, the detection of UTR70 was variable and often at the threshold of detection in both CD34+ and CD14+derived DCs (Fig. 1a, b). Furthermore, we could never detect RNA transcripts (UTR378) from the previously reported ip1 promoter in the reactivating DCs (data not shown). In contrast, robust MIEP-derived IE gene transcription was detected in the DCs suggesting that, in DCs at least, classical MIEP activity was required for IE gene expression (Fig. 1a, b).

Next, we performed the same analysis in THP1 cells stimulated with PMA to confirm we could re-capitulate previously published observations [17] and also to ensure we could detect UTR70 expression in our reactivation assays. In contrast to the DCs, robust ip2 derived transcription (UTR70) was observed alongside MIEP-derived transcription in these differentiated THP-1 cells (Fig. 1c).

### Impact of cell type and ligand-specific interactions on the origin of transcription

**Fig. 2.**
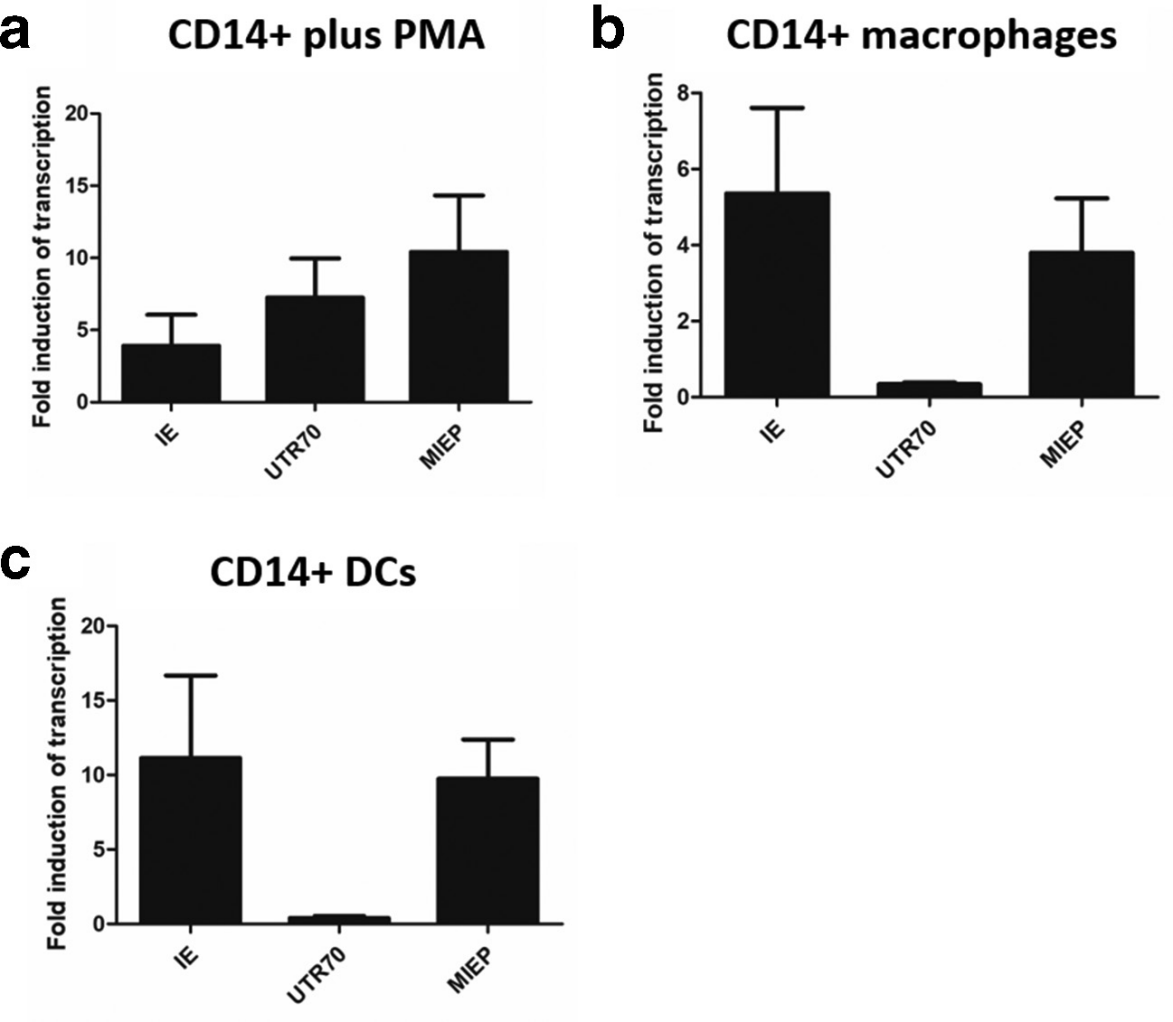
ip2-derived UTR70 transcripts are elevated in PMA stimulated monocytes (a–c). Experimentally latent CD14+ were incubated with PMA (a), IL-1/M-CSF to generate CD14+derived macrophages (b) or IL-4/GM-CSF/IL-6 to generate CD14+derived DCs (c) and RNA was analysed by qRT-PCR with primers that amplified UL123 (IE), MIEP-derived IE transcripts (MIEP), ip2-derived UTR70 transcripts (UTR70) and cellular 18S gene expression 24 h post stimulation with LPS. Gene expression is reported using the 2deltaCT method from two independent experiments except DCs where *n*=4.

It was possible that our experimental system of monocytes and their differentiation to DCs promoted the use of the MIEP in reactivation studies from experimental latency and thus we investigated the impact of stimulating monocytes down alternative differentiation pathways (Fig. 2). For the analysis we measured induction of total UL123 RNA expression alongside MIEP and ip2-derived (UTR70) transcripts. We observed that direct stimulation of CD14+monocytes with PMA resulted in both MIEP and ip2 IE-derived transcription (Fig. 2a). In contrast, differentiation with IL-1b/M-CSF to a macrophage phenotype resulted in a predominance of MIEP-derived transcripts (Fig. 2b). Again, differentiation of CD14+cells to DCs once again resulted in a predominance of MIEP-derived IE transcription during the early stages of viral reactivation (Fig. 2c). As expected, all stimulations of CD14+cells resulted in the transcription of UL123 RNAs (IE).

### Histone acetyltransferase inhibitors limit MIEP-derived IE transcription

These observations supported an inference of the previous study [17] that suggested the use of the alternative promoters to drive IE gene expression during reactivation may be dependent on cell type or ligand-specific activity (i.e. IE gene expression can, potentially, be activated by multiple cytokines via different signaling pathways). We have previously shown that chromatin modification plays a pivotal role in the regulation of HCMV latency [25] and, consistent with this, it is well documented that HDACi promote IE gene expression [26–32]. Most recently, we have reported that HATi prevent HCMV reactivation but noted that the individual HATi had cell-type-specific effects in DCs and THP-1-derived macrophages regarding their impact on IE gene expression in reactivating cells [21]. Specifically, the HATi C646, which targets the p300 family of HATs inhibited IE gene expression in DCs but not THP1 cells whereas the HATi MG149, which inhibits MOZ HAT activity prevented IE gene expression on both cell types.

**Fig. 3.**
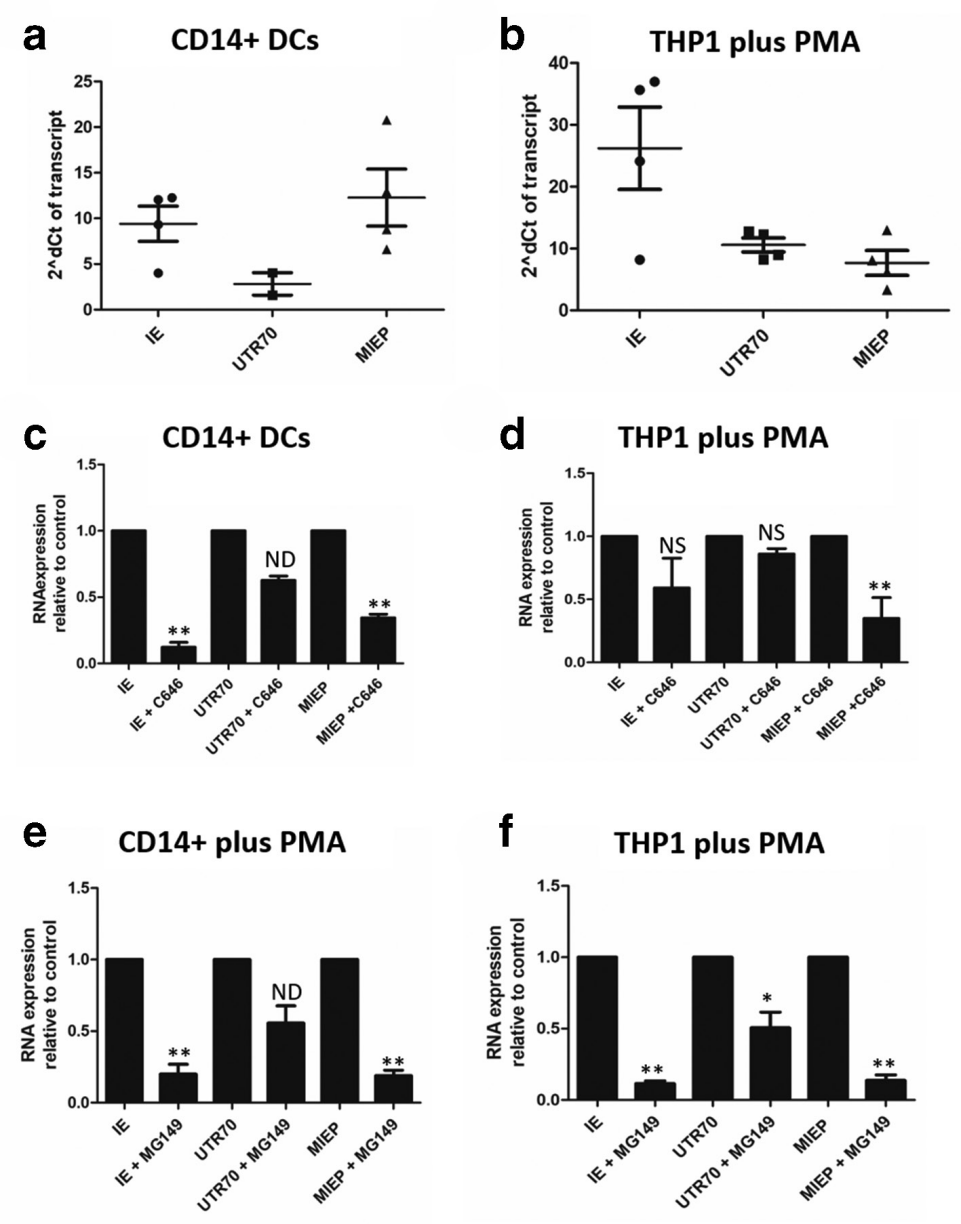
HATi display differential activity against the canonical and novel IE promoters (a–f). Experimentally latent immature CD14+derived DCs (a,c, e) or experimentally latent THP1 cells (b, d, f) were pre-incubated with DMSO (a–f) C646 (c,d) or MG149 (e,f) for 3 h then stimulated with IL-6 (a,c,e) or PMA (b,d,f). RNA was analysed by qRT-PCR with primers that amplified UL123 (IE), MIEP-derived IE transcripts (MIEP), ip2-derived UTR70 transcripts (UTR70) and cellular 18S gene expression 16 h post stimulation with IL-6. Gene expression is reported against immature cells for the control cells (a,b) to demonstrate baseline gene expression in controls. To measure impact of the HATi RNA expression is represented relative to RNA expression observed in the DMSO control, which is thus set at 1 (c–f) using the 2delta deltaCT method from three independent experiments. *P<0.05; **P<0.01; ns, non-significant; nd, insufficient data for analysis.

Consequently, we asked whether differences in the origin of IE transcription may explain the cell-type-specific effects of the HATi (Fig. 3). First we confirmed that UL123 RNA expression (IE) was detectable in both CD14+DCs and PMA-treated THP1 cells (Fig. 3a, b). As observed before (Fig. 1) MIEP-derived transcription predominated in CD14+derived DCs 16 h post IL-6 stimulation (Fig. 3a) whereas in PMA-treated THP1 cells both MIEP and ip2-derived transcription was detected (Fig. 3b). Having established the IE transcriptional profile in the control cells we examined IE transcription in C646- (Fig. 3c, d) and MG149- (Fig. 3e, f) treated cells. As shown previously, C646 potently inhibited IE transcription in CD14+DCs (Fig. 3c). Similarly, MIEP-derived IE transcription was inhibited by C646 in these cells. In contrast, in the experiments where UTR70-derived transcription was detected in the CD14+DCs (Fig. 3a), UTR70 expression in the presence of C646 was not significantly reduced (Fig. 3c). In the PMA-treated THP1 cell model, where we observed both MIEP- and ip2- (UTR70) derived transcripts (Fig. 3b), we again observed that C646 clearly reduced MIEP-derived IE gene expression in THP1 cells stimulated with PMA (Fig. 3d). However, the impact on the ip2-derived IE transcription (UTR70) was much less (Fig. 3d) suggesting that ip2 activity was largely independent of any p300-mediated activity in PMA-treated THP1 cells. Additionally, the levels of UL123 RNA expression were less effected by C646 in the PMA-treated THP1 cell type (Fig. 3d) compared to the effect in DCs (Fig. 3c), which was consistent with our prior report that C646 demonstrated CD14+DC-specific activity against IE gene expression [21].

In the same study that defined the cell-type-specific effect of the C646 HATi we reported that a second HATi, MG149, inhibited IE expression in both cell types [21]. MG149 targets the MYST family of HATs, and thus we assessed the impact of the MG149 inhibitor on MIEP- and ip2- (UTR70) derived transcripts. As seen previously [21], total IE gene expression was reduced in both CD14+derived DCs and PMA-treated THP1 cells in the presence of MG149 HATi (Fig. 3e, f). Although the impact of MG149 was most overt on the MIEP-derived transcription in both cell types (Fig. 3e, f). we did observe that the MG149 HATi also reduced levels of UTR70 transcripts in both THP1 cells and CD14+derived DCs. The effect on UTR70 expression was lower but the decrease in UTR70 expression was statistically significant (Fig. 3e, f).

### Latency reversing agents drive MIEP-derived transcription

**Fig. 4.**
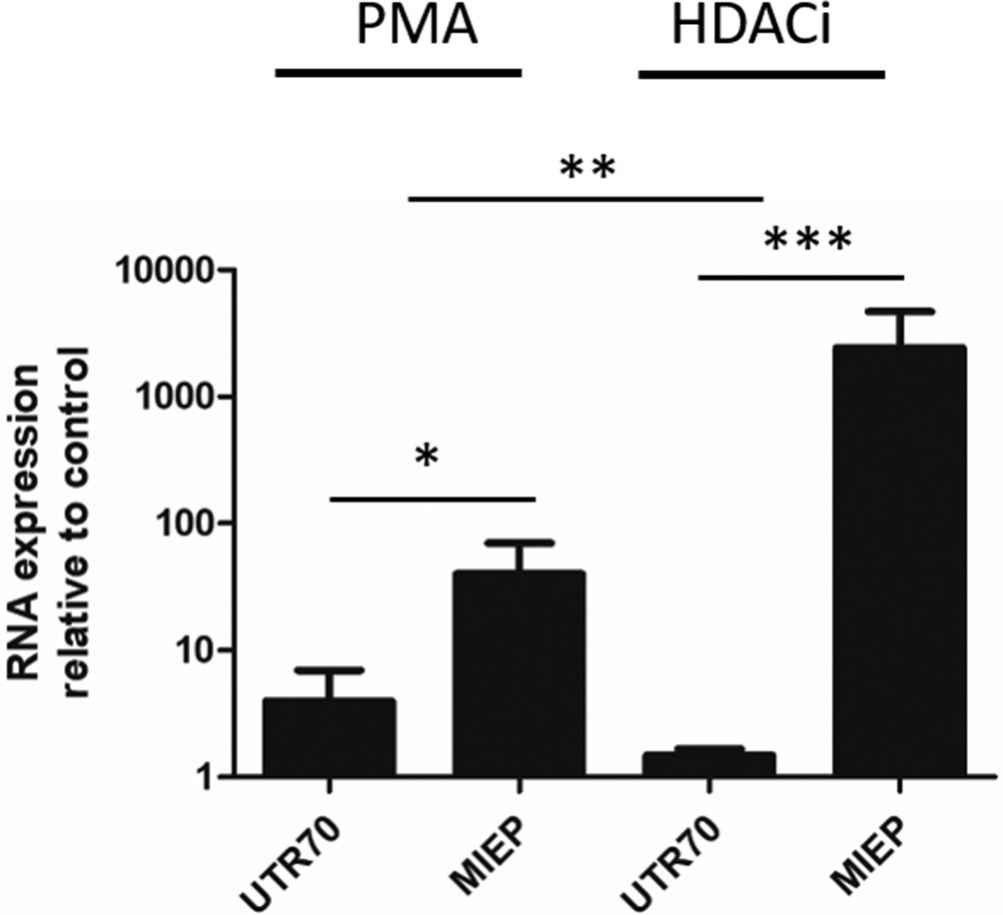
HDACi drive MIEP-derived transcription in experimentally latent CD14+cells. Experimentally latent CD14+cells were incubated with PMA or the HDACi Romdepsin for 24 h and then RNA analysed by qRT-PCR with primers that amplified MIEP-derived IE transcripts (MIEP), ip2-derived UTR70 transcripts (UTR70) and cellular 18S gene expression 24 h post stimulation. Gene expression is reported relative to the RNA expression observed in the unstimulated (for PMA) or DMSO controls (for HDACi) using the 2delta deltaCT method from three independent experiments. *P<0.05; **P<0.01; ***P<0.001.

One major aim of using chromatin reversing agents, such as HDACi to induce HCMV IE gene expression from latent virus, is to promote immune clearance of otherwise latently infected cells by so-called shock-and-kill approaches [28]. Thus, we assessed the impact of using known HDACi on IE transcription driven from the canonical MIEP and ip2 promoters (Fig. 4). Infected CD14+monocytes were incubated with either the HDACi Romidepsin or stimulated with PMA and total IE transcription alongside MIEP- and ip2-derived (UTR70) IE transcription measured. As seen before (Fig. 2a), PMA stimulation promoted both UTR70- and MIEP-derived IE transcript expression in CD14+cells when compared to background IE gene expression observed in these cells (Fig. 4). In contrast, the HDACi promoted robust MIEP derived IE expression (Fig. 4) with relatively little impact on UTR70 expression when analysed at 24 h post-addition of inhibitor. Indeed, the preferential activation of the MIEP over ip2 by HDACi was significantly different from the comparative responsiveness of these promoters to PMA stimulation (Fig. 4).

### UTR70 expression is sensitive to cycloheximide in CD14+derived DCs

**Fig. 5.**
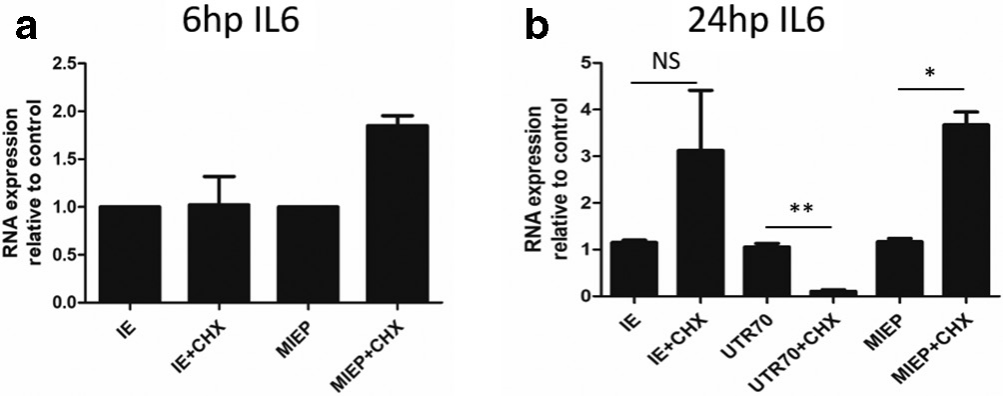
UTR70 gene expression is cycloheximide sensitive (a,b). Experimentally latent immature CD14+derived DCs were incubated with cycloheximide (CHX) or solvent control 6 h prior to incubation with IL-6 to promote reactivation. RNA was analysed by qRT-PCR with primers that amplified UL123 (IE), MIEP-derived IE transcripts (MIEP), ip2-derived UTR70 transcripts (UTR70) and cellular 18S gene expression at 6 (a) and 24 (b) h post reactivation with IL-6. Gene expression is reported relative to the RNA expression observed in the immature DCs using the 2delta deltaCT method from three independent experiments. *P<0.05; **P<0.01; NS=non-significant.

Our inability to consistently detect ip2-driven UTR70 transcription in reactivating DC samples led us to further investigate the regulation of the ip2-derived IE transcripts in these cells (Fig. 5). Samples of RNA from CD14+derived DCs stimulated with IL-6, with or without prior treatment (added 6 h prior to IL-6 addition) with protein synthesis inhibitor cycloheximide, were analysed for total IE expression as well as transcript origin by qPCR at 6 and 24 h post IL-6 treatment. The addition of cycloheximide had no impact on IL-6-induced IE gene expression in DCs (Fig. 5a) and, consistent with this, MIEP transcription was not impaired (Fig. 5a). At this timepoint of analysis, UTR70 expression was below the detection limit of the PCR and thus it was not possible to determine if cycloheximide had any impact on UTR70 expression. However, by 24 h post IL-6 treatment detectable levels of both MIEP- and UTR70-derived transcripts were detected. The accumulation of MIEP transcripts remained largely unaffected by cycloheximide and, indeed, appeared to be elevated (Fig. 5b). In contrast, pre-treatment with cycloheximide uniformly reduced UTR70 expression in all CD14+derived DCs tested (Fig. 5b).

## Discussion

The expression of viral IE gene products is crucial for lytic infection and their induction is considered pivotal for reactivation from latency. This concept holds true for all members of the herpesvirus family and thus the mechanisms that control the initiation of lytic infection or the molecular switch from latency to reactivation have been widely studied.

The prevailing hypothesis for HCMV has long been that the differential regulation of the viral MIEP, in a cell-type-specific manner, is the basis of MIEP regulation that dictates latency and reactivation. For the establishment of latency, the MIEP is required to be eventually silenced and thus, for reactivation to occur, the silencing of the MIEP must be reversed. This model is built on the view that host cell factors, involved in chromatin-mediated regulation of transcription, underpin this virus transcriptional control (reviewed in [1]). As such, in multiple *in vitro* models and, importantly, during natural latency, the MIEP is associated with methylated histones during latent infection and acetylated histones during the reactivation phase [12, 24, 26, 33, 34]. Indeed, it has been reported that UL138, a viral gene product expressed during latency, prevents the recruitment of histone demethylase activity to the MIEP, presumably to augment this silencing [35], strongly arguing that continued silencing of the MIEP in latency is central to the biology of HCMV. Further support for the key role chromatin plays in the regulation of HCMV latency are the observations that pharmacological manipulation of cells with histone modifying enzyme inhibitors promotes IE gene expression in latently infected CD34+ and CD14+cells [28]. The data presented here support this prevailing view that chromatin modification plays an important role in the regulation of the MIEP – HDACi inhibitors drive IE gene expression and inhibitors of HAT activity suppress the induction of IE gene expression in reactivating DCs. Taken together, this may suggest that the identification and validation of latency reversing agents that target chromatin to drive IE gene expression in a shock and kill approach is best achieved in primary CD34+ and CD14+models of latency.

Importantly, qPCR analyses suggest that the IE transcripts detectable in DCs differentiated from primary myeloid cell precursors are derived from the canonical MIEP. The data also suggest that the activity of the ip2 promoter is less sensitive to the activity of inhibitors of histone-modifying enzymes. Indeed, in the original study of ip2 activity, UTR70 transcription is evident in both latent and lytic infection of THP1 cells with PMA promoting an elevation of basal UTR70 RNA expression [17]. Indeed, it is intriguing that, in that study, the continued detection of UTR70 RNA transcripts in latently infected THP1 cells in long-term culture did not translate into continual IE protein production – IE protein was observed to rapidly decline 3 days post latency establishment in the same model [17]. Furthermore, the expression of UTR70 in latently infected cells is consistent with the original hypothesis of Arend *et al*. that the ip2 promoter is active in cells in which MIEP activity is limited – i.e. latently infected cells [18]. The detection of UTR70 transcription in an absence of IE protein production in the THP1 cells also introduces the possibility that post-transcriptional regulation of the UTR70 RNA could be occurring to prevent aberrant IE protein expression during latency. For example, both cellular and viral miRNAs have been suggested to play a role in the regulation of IE gene expression in myeloid progenitor cells and it is possible that one of their roles is to target these non-canonical transcripts [36–40].

The original identification of novel promoters that drive HCMV IE gene expression, which were non-essential for lytic infection, led to the logical consideration that they played a key role in HCMV reactivation. Intriguingly, a deletion of the ip2 promoter (and thus a deletion in the MIE locus) was observed to generate a reactivation defect in latently infected THP1 cells and a CD34+model system of latency [17]. Indeed, in our analyses, we also observed the expression of UTR70 (under the control of ip2) in latently infected THP1 cells although we also could detect MIEP-derived IE transcripts in these cells as well. However, in DCs derived from CD34+ and CD14+precursors, we detected little evidence of UTR70 transcripts – in contrast to readily detectable MIEP-derived transcripts, which also correlated directly with the IE transcription phenotype. Thus, at least in DCs, IE gene expression appears to correlate with classical MIEP activity.

Our data in no way dismisses a role for the non-canonical IE promoters. Indeed, we clearly observe ip2 driven UTR70 transcripts, particularly in THP1 cells and also in primary monocytes stimulated with PMA. Furthermore, it is well established that the MIEP is subject to complex patterns of regulation underpinned by the cell type used. For example, deletion of the CREB response elements from the proximal canonical MIEP resulted in a defect in HCMV reactivation in DCs whereas a HCMV virus with NF-kB site MIEP deletions was less affected [16]. However, in other models of reactivation from latency, a clear phenotype with NF-kB MIEP deletions has been observed [41–43]. Most recently, blockade of AP1 activity has also been demonstrated to impact on HCMV MIEP-driven gene expression in both the Kasumi 3 and a CD34+model of latency and reactivation [13]. Indeed, an earlier study suggested that AP1 binding sites are crucial for HCMV reactivation in the context of NF-kB activity [20]. It is also worth saying that the identity of the differentiated CD34+cells grown on feeder cells used by Goodrum and colleagues is unreported [44] but it is possible that these are more macrophage-like compared to the DCs derived from CD34+cells [45]; this may explain why, in these differentiated CD34+cells, major IE promoter usage is more akin to that seen in differentiated macrophage-like THP1 cells. If this is the case, then it may point towards the exciting possibility of cell-type-specific roles for specific transcription factor binding sites within the MIEP and, additionally, alternative MIE promoters in HCMV reactivation that expands the complexity of HCMV reactivation. Certainly, from an evolutionary standpoint, an ability to re-initiate IE gene expression under multiple conditions would represent a more efficient basis for driving reactivation – an event that likely underpins transmission and consequent high seroprevalence of HCMV in the population.

We accept that in this study that we have not directly assessed the impact of the ip2 deletion virus on reactivation of HCMV in DCs. However, our data clearly show that in CD34+ or monocyte derived DCs the origin of the IE transcription is derived from the canonical MIEP derived and is not inhibited by cycloheximide. In contrast, we note that, when detectable in our hands, ip2-derived IE transcription in DCs was cycloheximide dependent, which would be consistent with it being considered to be an early/late lytic promoter as suggested in the original studies in fibroblasts [18]. That said, we do acknowledge a caveat to the cycloheximide analysis in that, whilst suggestive, it is not definitive and we remain cautious in our interpretation of the cycloheximide block experiments. For instance, although cycloheximide sensitivity is considered a marker of an early/late gene, in the original study of herpes virus temporal gene expression, cycloheximide release experiments were actually required to identify the major IE protein ICP0 of HSV [46]. Furthermore, in studies of baculovirus gene-expression kinetics, cycloheximide has been shown to have clear concentration-specific effects that complicated the temporal classification of virally expressed genes [47]. Here we observed that cycloheximide treatment leads to elevated HCMV MIEP transcription over time when compared to controls – again this enhancing effect of cycloheximide on IE gene expression has also been reported in studies of HSV [46, 48, 49]. It was postulated that a loss of early genes that repress IE transcription may partially explain this phenomenon in HSV [48]. In the case of HCMV this represents a possible explanation since translation of the IE RNA is blocked by cycloheximide and thus the MIEP auto-regulatory activity of IE86 (directed against the *cis* repression sequence present in the MIEP [30, 50]) is not active due to a lack of IE86 protein synthesis.

What is not clear is whether the ip2 promoter is required for lytic infection of differentiated THP1 cells; if this was the case, this would partially explain the importance of ip2 activity for viral replication upon reactivation in these cells as major IE gene expression represents the only first step towards viral reactivation. Additionally, it will be important to investigate the very plausible possibility that this is not an either/or scenario. We noted that in the studies of the ip2 deletion virus, a defect in MIEP-derived transcription was also evident upon reactivation in the CD34+cells [17]. It is also not clear what impact large deletions of the viral genome in the MIE region would have on the normal regulation of the MIEP and thus, even in these deletion viruses, regulatory events acting on the MIEP may still be important. For example, it has been demonstrated that an important CTCF binding site is present in the first intron of the MIE locus (and thus downstream of the MIEP), which has important regulatory functions for IE gene expression via an ability to modulate canonical MIEP activity [51].

In summary, we report that we observe that alternative ip2-driven IE transcription (generating UTR70 transcripts) clearly occurs in the THP1 cell model of HCMV latency and reactivation and also is evident in CD14+cells treated with phorbol ester (PMA) and this is entirely consistent with the previous identification of a novel reactivation associated IE promoter [17]. However, in CD34+ or CD14+derived DCs as well as CD14+derived macrophages, MIEP-derived transcription predominates upon induction of virus reactivation – cell types that are established sites of reactivation for natural latency. Furthermore, pharmacological stimulation of CD14+cells with HDAC inhibitors promotes IE transcription from the canonical MIEP congruent with a role for chromatin as a key regulator of IE gene expression during HCMV latency and reactivation. Finally, we observe that ip2-driven UTR70 transcripts and canonical MIEP-derived IE transcripts display differential sensitivity to cycloheximide upon reactivation in DCs. These data, together with published studies of viruses with mutations in the MIEP, argue that the canonical MIEP retains an important role in HCMV reactivation in DCs.
